# Stereotactic IMRT for prostate cancer: Dosimetric impact of multileaf collimator leaf width in the treatment of prostate cancer with IMRT

**DOI:** 10.1120/jacmp.v5i2.1989

**Published:** 2004-08-16

**Authors:** L. Wang, B. Movsas, R. Jacob, E. Fourkal, L. Chen, R. Price, S. Feigenberg, A. Konski, A. Pollack, C. Ma

**Affiliations:** ^1^ Department of Radiation Oncology Fox Chase Cancer Center Philadelphia Pennsylvania U.S.A. 19111

**Keywords:** IMRT, mMLC, prostate cancer, radiation dosimetry

## Abstract

The focus of this work is the dosimetric impact of multileaf collimator (MLC) leaf width on the treatment of prostate cancer with intensity‐modulated radiation therapy (IMRT). Ten patients with prostate cancer were planned for IMRT delivery using two different MLC leaf widths—4 mm and 10 mm— representing the Radionics micro‐multileaf collimator (mMLC) and Siemens MLC, respectively. Treatment planning was performed on the XKnifeRT2 treatment‐planning system (Radionics, Burlington, MA). All beams and optimization parameters were identical for the mMLC and MLC plans. All the plans were normalized to ensure that 95% of the planning target volume (PTV) received 100% of the prescribed dose. The differences in dose distribution between the two different plans were assessed by dose–volume histogram (DVH) analysis of the target and critical organs. We specifically compared the volume of rectum receiving 40 Gy $\left(V_{40}\right)$, 50 Gy $\left(V_{50}\right)$, 60 Gy $\left(V_{60}\right)$, the dose received by 17% and 35% of rectum ($D_{17}$ and $D_{35}$), and the maximum dose to 1 cm^3^ of the rectum for a prescription dose of 74 Gy. For the urinary bladder, the dose received by 25% of bladder $\left(D_{25}\right)$, $V_{40}$, and the maximum dose to 1 cm^3^ of the organ were recorded. For PTV we compared the maximum dose to the “hottest” 1 cm^3^ $\left(D_{\max1\text{cm}3}\right)$ and the dose to 99% of the PTV $\left(D_{99}\right)$. The dose inhomogeneity in the target, defined as the ratio of the difference in $D_{\max1\text{cm}3}$ and $D_{99}$ to the prescribed dose, was also compared between the two plans. In all cases studied, significant reductions in the volume of rectum receiving doses less than 65 Gy were seen using the mMLC. The average decrease in the volume of the rectum receiving 40 Gy, 50 Gy, and 60 Gy using the mMLC plans was 40.2%, 33.4%, and 17.7%, respectively, with p<0.0001 for $V_{40}$ and $V_{50}$ and p<0.012 for $V_{60}$. The mean dose reductions for $D_{17}$ and $D_{35}$ for the rectum using the mMLC were 20.4% (p<0.0001) and 18.3% (p<0.0002), respectively. There were consistent reductions in all dose indices studied for the bladder. The target dose inhomogeneity was improved in the mMLC plans by an average of 29%. In the high‐dose range, there was no significant difference in the dose deposited in the “hottest” 1 cm^3^ of the rectum between the two plans for all cases (p>0.78). In conclusion, the use of the mMLC for IMRT of the prostate resulted in significant improvement in the DVH parameters of the prostate and critical organs, which may improve the therapeutic ratio.

PACS number: 87.53.Tf

## I. INTRODUCTION

The multileaf collimator (MLC) has been increasingly used in radiotherapy to facilitate delivery of three‐dimensional conformal radiotherapy (3D‐CRT) and intensity‐modulated radiation therapy (IMRT). The main goals of these techniques are to minimize dose to the normal tissues that surround the target and to improve the conformality of dose to the tumor enabling a reduction in the irradiated volume. As the primary device used to shape the radiation fields, the leaf width of a MLC device could be of importance. Most commercial MLCs have a leaf width of 1.0 cm defined at the isocenter. With such a leaf width, conformation of the field to an irregularly shaped target may be poor in comparison with mMLC and blocks. The dosimetric effects of the 1.0‐cm leaf width multileaf collimators have been described previously.^(^ ^1^ ^–^ ^3^ ^)^ The limitation of a broad‐leaved MLC may be pronounced when employing small fields in stereotactic radiotherapy or treating targets in close proximity to critical organs.

Smaller leaf width MLCs, commonly called “micro” or “mini”‐MLCs (mMLC), with leaf widths between 1.6 mm and 5 mm defined at the plane of isocenter, were designed to overcome these potential limitations.^(^ ^4^ ^–^ ^7^ ^)^ A study on the impact of collimator leaf width in stereotactic radiosurgery for brain tumors and 3D‐CRT for prostate cancer using 1.7‐mm, 3‐mm, and 10‐mm leaf width MLCs was first reported by Kubo et al.^(8)^ They found that the use of a mMLC with leaf widths of 1.7 mm to 3.0 mm allowed them to improve the ratios between the volume encompassed by the prescription isodose surface and the clinical target volume, thus reducing the dose to the surrounding normal tissue. Another study of the effect of MLC leaf width on IMRT was performed by Fiveash et al.^(9)^ They compared physical dose distributions resulting from a 5‐mm and a 10‐mm MLC in the treatment of central nervous system and head and neck neoplasms. Their study demonstrated an improved physical dose distribution using the 5‐mm MLC for brain and head and neck IMRT.

Since prostate cancer is a dose‐responsive neoplasm,^(10)^ increasing the prescription dose has been shown to improve local control of carcinoma of the prostate.^(^ ^11^ ^–^ ^15^ ^)^ However, dose escalation has been limited due to injuries to the rectum. Although 3D‐CRT and IMRT have reduced the morbidity of prostate radiotherapy, the incidence of late rectal bleeding/injury has been shown to increase as the prescription dose rises above 70 Gy.^(16)^ ^,^ ^(17)^ Several studies^(^ ^16^ ^–^ ^21^ ^)^ have also indicated that volume effects may influence the incidence of chronic rectal injuries. The percentage volume of the rectum exposed to doses between 40 Gy and 50 Gy and the existence of a reserve of unexposed tissue also play a role in the development of late rectal injury.^(16)^ These studies suggest that late rectal bleeding/injury is a dose‐limiting complication in prostate radiotherapy. Therefore, it is important to explore our ability to maximize the radiation dose to the target while minimizing dose to the surrounding critical organs. This is especially important in the stereotactic IMRT approach in which higher radiation doses, or dose escalation, will be utilized. By studying the dosimetric impact of two MLCs of different leaf widths (4 mm versus 10 mm) in the treatment of prostate cancer with IMRT, we will be able to determine quantitatively the dosimetric superiority of a narrow‐leaf MLC, and thus improve our ability to delivery optimal radiation dose in stereotactic IMRT for prostate radiotherapy.

## II. METHODS

All treatment plans for this study were generated on the XKnifeRT2 treatment‐planning system (TPS) (Radionics, Burlington, MA). This system is designed specifically for stereotactic conformal and IMRT planning employing a mMLC with a leaf width of 4 mm at the isocenter. The mMLC device is attached to the head of the linear accelerator prior to use and has a maximum field size of 13.4 cm×10.8 cm. The TPS also allows for treatment planning for delivery using the Siemens MLC (Siemens, CA) with a leaf width of 10 mm, once beam data for the corresponding MLC are collected and commissioned. The characteristics of the Siemens MLC and the Radionics mMLC, such as leaf leakage, scatter, and tongue‐and‐groove effect, are not directly modeled in the inverse treatment planning. However, the MLC tongue‐and‐groove effect on IMRT dose distributions is known to be small^(22)^ or clinically negligible for the composite plan.^(23)^ The effect of leaf‐end is modeled by using the leaf offset, which accounts for the poor penumbra of the curved leaf ends. For both MLCs, the leaf offset is the same. The difference in beam commissioning for two different MLCs is the penumbra modeling based on penumbra measurement. The measured penumbra for the MLC and the mMLC differs only by 1 mm. Also, there are no significant mechanical limitations for the mMLC and Siemens MLC, except the leaf width.

### A. Target definition and plan preparation

The IMRT plans for actual treatment were generated from the Corvus planning system (Nomos Corp, Sewickley, PA) using either 6 MV or 10 MV. For this comparative dosimetric study, a second CT scan was obtained for each patient (with IRB approval) who was immobilized in a customized body cast in the supine position. A stereotactic body frame was affixed to the base board to which the body cast was also attached. The body frame was used to establish a rigid coordinate system, which is required for the TPS.

Patients were scanned at 3‐mm slice intervals from the proximal femur to 1.5 cm above the iliac crest. After the CT images were transferred into the TPS, the body localizer rods were registered in order to proceed to contouring and treatment planning. The clinical target volume (CTV)^(24)^ was the prostate gland, which was outlined by the physician. The rectum, including cavity, was contoured from the bottom of the ischial tuberosity to the recto‐sigmoid junction. The average volume of rectum is 78.7 cm^3^, ranging from 43.5 cm^3^ to 121.5 cm^3^. The other critical organ contoured was the whole bladder. The PTV ^(24)^ was generated from the CTV, using a margin of 8 mm in all directions (e.g., anterior, lateral, superior, and inferior), except in the posterior direction, where a 4‐mm margin was used. These margins are the same as those used for clinical IMRT planning in our institution based on using a BAT (B‐mode Acquisition and Targeting, Normos Corp. Sewickley, PA) ultrasound system for daily localization. The average PTV volume is 156.2 cm^3^, ranging from 75.7 cm^3^ to 262.7 cm^3^. The expansion of the CTV to PTV resulted in some overlaps with the rectum and the bladder. In order to ensure adequate coverage of the PTV, an extra margin of 2 mm was defined around the PTV to provide an expanded volume to use as the “target” during optimization.^(25)^

We used 6 MV photons for all treatment plans, since this is the only beam generated from the Siemens Primart accelerator, upon which the mMLC is mounted for stereotactic conformal and step‐and‐shoot IMRT delivery. For all treatment plans the number of beams, gantry positions, and collimator angles were taken from clinically treated coplanar IMRT plans using 6 to 9 fields. A prescription dose of 74 Gy in 37 fractions was used for all patients in this comparison study.

### B. Inverse treatment planning

Inverse treatment planning on the XKnifeRT2 TPS employs a dose gradient method for optimization. The inverse optimization was performed based on an objective function defined as the weighted quadratic difference of the prescribed and calculated dose distribution. The details of the algorithm can be found elsewhere.^(^ ^26^ ^–^ ^28^ ^)^ The IMRT planning involved setting up the plan optimization parameters and dose constraints. These constraints include maximum and minimum doses for the target and critical organs and the penalties for violating each. We manually optimized these plan specification parameters as a compromise between target coverage and protection of the rectal wall and bladder for each individual patient. For each patient, the same optimization parameters were used for both mMLC and MLC plans.

The output of the optimization process was an “ideal” intensity distribution for each beam. The theoretically optimized intensity profiles were then “stratified” by the built‐in leaf sequencer based on the number of intensity levels selected in order to generate deliverable sequences of MLC segments and the leaf designs. Having compared the effects of the intensity level on the mMLC plans and the MLC plans, we adopted seven intensity levels for both mMLC and MLC plans. It was found that further increasing the number of intensity levels resulted in a negligible benefit. The use of the same intensity levels is intended to eliminate another variable that may affect the way of leaf sequencing.

### C. Plan comparison

The final dose calculations, using the stratified deliverable intensity distributions, were performed based on a measurement‐based pencil beam algorithm with inhomogeneity correction. We used 2‐mm dose‐grid resolution to construct dose–volume histograms (DVHs) for plan comparison. All plans were normalized based on the DVHs to ensure that 95% of the PTV received 100% of the prescribed dose. Plans were deemed acceptable if they satisfied the rectal and bladder criteria used in our department to treat prostate cancer patients. Specifically, rectal criteria require that no more than 17% of the rectum should receive more than 65 Gy, and no more than 35% of the rectum should receive greater than 40 Gy. For the bladder, we required that no more than 25% of the bladder should receive greater than 65 Gy; and no more than 50% of the bladder should receive greater than 40 Gy. All the plans generated for this study satisfied these criteria.

The differences in radiation dose distribution between the two different MLC plans were assessed based on the DVHs of the target and critical organs for each patient. Since the maximum and minimum doses were determined by a few points (a few voxels), they might be misleading measures of dosimetric differences. Thus, in our study, the maximum dose was defined as the maximum dose received by the “hottest” 1 cm^3^ in the volume of interest $\left(D_{\max1\;\text{cm}3}\right)$, while the minimum dose was defined as the dose to 99% of the PTV $\left(D_{99}\right)$. The dose inhomogeneity was defined as the ratio of the difference between $D_{\max1\;\text{cm}3}$ and $D_{99}$ to the prescribed dose. Since the CTV was completely inside the PTV, the coverage and dosimetric characteristics were less affected by the size of the leaf width; hence, the dose parameters for the CTV were not compared. For the rectum, $D_{\max1\;\text{cm}3}$ and doses received by 17% $\left(D_{17}\right)$ and 35% $\left(D_{35}\right)$ of the rectum were compared. The volumes receiving 40 Gy $\left(V_{40}\right)$, 50 Gy $\left(V_{50}\right)$, and 60 Gy $\left(V_{60}\right)$ were also compared to demonstrate the overall distribution of the DVH. For the bladder, the dose received by 25% $\left(D_{25}\right)$ of the bladder and the volume receiving 40 Gy $\left(V_{40}\right)$, as well as $D_{\max1\text{cm}3}$ were compared.

We calculated the percentage differences between the two MLC plans for the dose indices described above. To determine whether the differences between the two groups of plans were statistically significant, we used the paired *t*‐test to measure the differences. A *p*‐value was determined for each dose index compared using a two‐tailed test and a degree of freedom of 18.

## III. RESULTS

### A. PTV doses

**Figure 1:**
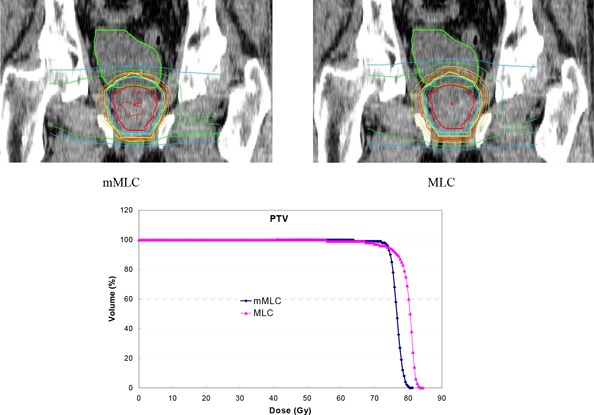
Comparison of a typical isodose distribution on the coronal view and a DVH of the PTV between the MLC‐ and mMLC‐based plans.

**Figure 2:**
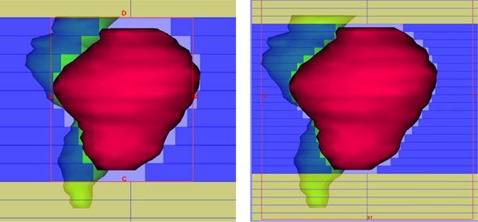
Illustration of the impact of leaf width on beam shaping through the comparison of the beam's‐eye‐view.

All plans were normalized based on the DVH to ensure that 95% of the PTV was covered by the prescription dose; therefore, the $D_{95}$ for both mMLC and MLC plans were identical. Table 1 lists the $D_{\max1\text{cm}3}$ and $D_{99}$ and dose inhomogeneity for both mMLC and MLC based plans for each patient. The ratios between the mMLC and MLC plans for these parameters are tabulated next to the individual parameter for each patient. It is seen that the ratios for $D_{99}$ are consistently higher than unity, indicating that the mMLC‐based plans consistently resulted in a higher minimum dose $\left(D_{99}\right)$. The average difference in $D_{99}$ of the PTV is 3%±0.02% (p<0.0002), with the averages being 72.20 Gy for the mMLC plans and 69.96 Gy for the MLC plans. For $D_{\max1\text{cm}3}$, the ratios are less than unity, except for one case, indicating that the mMLC‐based plans generally resulted in a lower maximum dose $\left(D_{\max1\text{cm}3}\right)$. The average $D_{\max1\text{cm}3}$ of the PTV was 80.5 Gy for the mMLC plans and 82.3 Gy for the MLC plans. Although differences in $D_{\max1\text{cm}3}$ for the PTV were small, it was statistically significant (p<0.002). The increased minimum doses and the reduced maximum doses associated with the mMLC implied improved dose homogeneity, as shown in the last column by the ratio of dose inhomogeneity between mMLC and MLC. The average PTV dose inhomogeneity was 11.3% and 16.6% for the mMLC plans and the MLC plans, respectively. A typical isodose distribution and DVH comparison for the PTV are shown in Fig. 1, where the characteristic difference observed between the mMLC plans and the MLC plans for all of the patients is demonstrated. To illustrate the dosimetric impact of the leaf width of the mMLC, a comparison of the beam's‐eye‐view is presented in the Fig. 2. It is apparent that larger leaf width results in poorer beam conformality, leading to increased irradiation to the surrounding normal tissues.

**Table 1:** The maximum and minimum doses and dose inhomogeneities of the PTV for each patient compared between the mMLC‐based and the MLC‐based plans, respectively.

	$D_{\max1\text{cc}}$ (Gy)			$D_{99}$ (Gy)			Dose inhomogeneity		
Patient	mMLC	MLC	ratio	mMLC	MLC	ratio	mMLC	MLC	ratio
# 1	81.40	84.40	0.96	71.30	68.50	1.041	13.65%	21.49%	0.635
# 2	80.00	82.90	0.97	71.64	67.14	1.067	11.30%	21.30%	0.530
# 3	79.70	82.70	0.96	72.97	70.10	1.041	9.09%	17.03%	0.534
# 4	82.30	81.70	1.01	73.10	71.70	1.020	12.43%	13.51%	0.920
# 5	80.60	81.50	0.99	71.95	70.10	1.026	11.69%	15.41%	0.759
# 6	80.55	80.70	1.00	72.89	71.30	1.022	10.35%	12.70%	0.815
# 7	81.70	84.20	0.97	71.94	71.00	1.013	13.19%	17.84%	0.739
# 8	80.30	81.80	0.98	72.60	69.58	1.043	10.41%	16.51%	0.630
# 9	79.20	80.50	0.98	73.00	71.20	1.025	8.38%	12.57%	0.667
# 10	79.84	82.50	0.97	70.60	69.00	1.023	12.49%	18.24%	0.684
**Average**	**80.56**	**82.29**	**0.98**	**72.20**	**69.96**	**1.032**	**11.30%**	**16.66%**	**0.69**

### B. Rectal Doses

**Figure 3:**
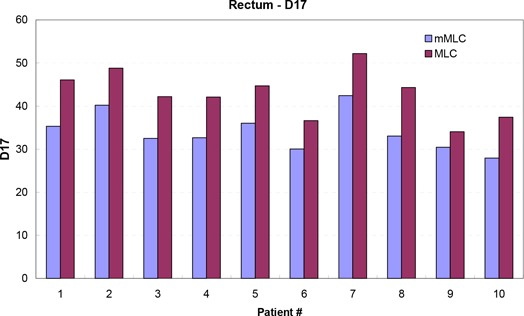
Comparison of the dose received by 17% $\left(D_{17}\right)$ of the rectum between the mMLC‐ and MLC‐based plans.

**Figure 4:**
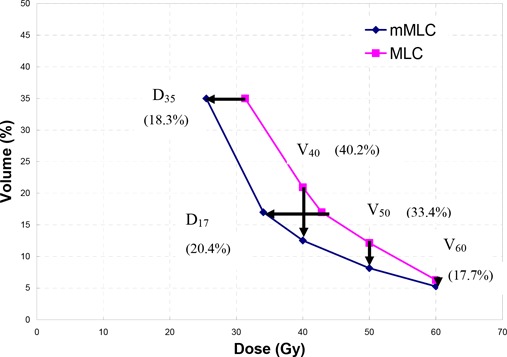
The average dose indices resulting from the two types of multileaf collimator treatment planning. The associated percentage increase from MLC to mMLC for each parameter is presented next to the dose parameter of interest.

Figure 3 compares the $D_{17}$ of the rectum in histograms between the two plans on a per patient basis. Similar differences and statistics were found for other dose indices ($D_{35}$, $V_{40}$, $V_{50}$, and $V_{60}$), which are tabulated in Table 2. The mean improvement in the $D_{17}$ and $D_{35}$ using the mMLC plans was 20.4%±5.0% (p<0.0001) and 18.3%±9.0% (p<0.0002), respectively. The average $V_{40}$, $V_{50}$, and $V_{60}$ are 12.42%, 8.03%, and 5.13% for the mMLC plans, respectively, and 20.81%, 12.01%, and 6.16% for the MLC plans. This corresponds to average volume reductions of 40%±9.0% (p<0.0001), 33%±10.0% (p<0.0001), and 18%±17.0% (p<0.012) receiving doses of 40 Gy, 50 Gy, and 60 Gy, respectively. Figure 4 compares the mean values of $D_{17}$, $D_{35}$, $V_{40}$, $V_{50}$, and $V_{60}$ averaged over the 10 patients between the mMLC and MLC plans to illustrate a common trend of the rectal DVH.

**Table 2:** The dose indices $\left(D_{17},D_{35},V_{40},V_{50},V_{60}\right)$ of the rectum for each patient compared between the mMLC‐ based and the MLC‐based plans, respectively.

Patient	$D_{17}$		$D_{35}$		$V_{40}$		$V_{50}$		$V_{60}$	
	mMLC	MLC	mMLC	MLC	mMLC	MLC	mMLC	MLC	mMLC	MLC
# 1	35.30	46.07	20.30	27.34	14.50	21.80	10.50	14.70	7.40	8.80
# 2	40.20	48.78	31.20	37.03	17.00	28.80	11.00	15.80	6.90	7.70
# 3	32.50	42.20	24.95	32.07	10.20	20.40	7.00	9.30	4.30	4.30
# 4	32.64	42.10	25.84	31.40	10.80	19.30	7.30	11.60	5.10	6.50
# 5	36.03	44.70	27.30	30.05	14.00	21.10	9.30	13.00	5.60	6.70
# 6	29.88	35.97	25.56	27.90	6.00	12.50	3.20	6.00	1.30	2.40
# 7	42.42	52.20	31.85	40.50	20.10	36.10	10.90	19.60	6.50	10.00
# 8	33.02	44.30	24.70	30.05	12.40	20.60	8.20	12.70	5.60	6.30
# 9	30.44	34.05	21.65	34.05	10.00	12.30	6.30	7.80	4.40	4.00
# 10	27.92	37.40	21.78	23.88	9.20	15.20	6.60	9.60	4.20	4.90
**Average**	**34.04**	**42.78**	**25.51**	**31.43**	**12.42**	**20.81**	**8.03**	**12.01**	**5.13**	**6.16**

**Figure 5:**
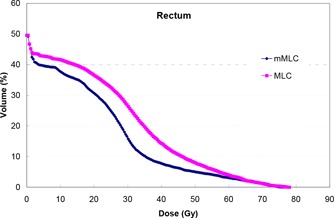
A typical comparison of the rectum DHV between the mMLC‐ and MLC‐based plans.

In the high‐dose region, the PTV usually overlapped the rectum. For this portion of the rectum, a compromise was reached between the target coverage and normal tissue sparing. Figure 5 shows a typical comparison of rectal DVHs of the mMLC and the MLC plans. Since we normalized plans to make the $D_{95}$ of the PTV equal in both plans, the colder spot in the PTV (represented by $D_{99}$) with the MLC plans (as seen in Fig. 1) led to a reduced maximum dose to the rectum, compared to that obtained with the mMLC plans. If target coverage has the highest priority, a new normalization value would be selected (e.g., making $D_{99}$ of the PTV equal in both plans to ensure the same coverage). Under the condition that the same degree of coverage was ensured for the PTVs, the average $D_{\max1\text{cm}3}$ of the rectum for the two group of plans was found not significantly different (p>0.7), with an average of 75.10 Gy for the mMLC plans and 74.93 Gy for the MLC plans.

### C. Bladder dose

**Figure 6:**
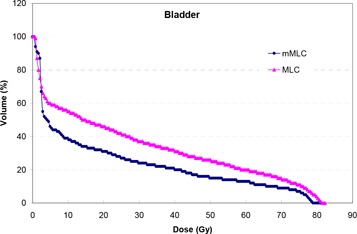
A typical comparison of the bladder DHV between the mMLC‐ and MLC‐based plans.

Table 3 compares the dose indices of $D_{25}$, $V_{40}$, and the $D_{\max1\text{cm}3}$ for the bladder between the mMLC‐ and MLC‐based plans. A typical comparison of bladder DVHs is presented in Fig. 6. It is seen in Table 3 that the mMLC offers a consistent dose reduction in terms of $D_{25}$, $V_{40}$, and $D_{\max1\text{cm}3}$ for the bladder, the average values of dose reduction being 32.2% (p<0.0001), 25.0% (p<0.0001), and 1.2% (p<0.08), respectively.

**Table 3:** The dose indices ($D_{25}$, $V_{40}$, and $D_{\max1\text{cc}}$) of the bladder for each patient compared between the mMLC‐based and the MLC‐based plans, respectively.

Patient	$D_{25}$		$V_{40}$		$D_{\max1\text{cc}}$	
	mMLC	MLC	mMLC	MLC	mMLC	MLC
# 1	18.80	28.02	11.90	17.60	79.50	80.23
# 2	29.70	51.69	20.30	31.80	78.80	81.20
# 3	4.30	16.80	6.60	11.10	77.80	79.80
# 4	4.08	15.23	9.60	11.30	76.83	77.20
# 5	48.90	55.00	34.20	39.70	77.50	79.50
# 6	25.60	29.20	10.50	13.30	79.30	78.66
# 7	48.06	53.95	33.00	40.60	79.70	82.90
# 8	40.40	49.50	25.30	33.60	79.00	77.10
# 9	45.05	51.70	29.60	35.00	78.60	79.20
# 10	34.60	51.92	21.80	31.90	78.80	79.42
**Average**	**29.95**	**40.30**	**20.28**	**26.59**	**78.58**	**79.52**

## IV. DISCUSSION

It is intuitive that a smaller leaf width should result in better beam shaping, leading to improved dose conformality to the target and lower doses to surrounding normal tissue. However, it is possible that the gains from a smaller leaf width using IMRT may not be clinically meaningful. Our results document significant dosimetric improvement using the mMLC. These dosimetric improvements may reduce chronic rectal injuries, as suggested from several studies.^(16)^ ^,^ ^(17)^ ^,^ ^(29)^ These studies had shown that rectal bleeding and late rectal toxicity are significantly correlated with the absolute/percentage volume of the rectum receiving all ranges of dose.

For the PTV, the reduced maximum dose and improved dose homogeneity may benefit prostate cancer patients because the prostatic urethra is within the treatment target and high dose may lead to higher acute and chronic injuries. In prostate brachytherapy, increased doses to the urethra have correlated with increased acute and chronic urinary symptoms.^(30)^ ^,^ ^(31)^ Certainly, the inhomogeneity in brachytherapy is considerably higher than that seen with external radiation, and a small improvement in homogeneity is probably not clinically significant.

In this study, patient positioning uncertainty is implicitly assumed to be the same, because we have used the same margin (8 mm) for both MLC‐ and mMLC‐based treatment planning. If the uncertainty in patient positioning can be reduced further, for example, by using a real‐time imaging system, treatment margins may also be reduced. This reduction of margin leads to a smaller PTV, in which case, a mMLC is even more beneficial because of its higher resolution.

The dosimetric differences reported here are believed to be solely due to the different leaf widths used in the treatment planning, since our comparisons were performed on the same treatment‐planning system using the same beam configurations, optimization parameters, and dose constraints for each patient. It is possible that the absolute dose indices may differ and the magnitudes of the differences between the mMLC and MLC plans may vary if a different treatment planning optimization system is used. However, the general findings of this study are expected to hold. It was also noted that the dosimetric difference was not caused solely by the leaf penumbra, but mainly by an effective penumbra due to the leaf resolution (width), since the difference in the penumbra is small. The leaf resolution determines the beamlet size, which has the most influence on the dosimetric performance.

Our results showed that smaller leaf width results in more MLC segments to deliver the prescribed dose; consequently, it required more monitor units for the treatment. The number of segments and monitor units was increased by an average of 22% with the mMLC compared to that with the MLC. One should rely on clinical judgment to decide whether such increased treatment time can be justified by the improved target coverage and significant dose reduction to the critical structures, such as rectum and bladder.

## V. CONCLUSIONS

This paper describes the quantitative dosimetric difference in treatment plans using two different leaf widths for IMRT delivery for prostate cancer. For all 10 patients planned in the XKnifeRT it was found that the dose uniformity of the planning target was improved substantially (by an average of 29%) by using the mMLC. There were significant reductions in critical organ volumes irradiated to high doses using the mMLC. Therefore, we conclude that the 4‐mm multileaf collimator offers a clinically significant improvement in critical organ protection over the conventional 1.0‐cm MLC in the treatment of prostate cancer using IMRT planned with this TPS, while simultaneously ensuring improved target dose coverage. Because of its superior ability to spare normal tissue, the use of a small leaf width MLC may improve the therapeutic ratio by reducing toxicity to adjacent critical organs during IMRT delivery. It is also ideal for dose escalation using hypo‐fractionation.
